# Evolutionary Modeling and Prediction of Non-Coding RNAs in *Drosophila*

**DOI:** 10.1371/journal.pone.0006478

**Published:** 2009-08-11

**Authors:** Robert K. Bradley, Andrew V. Uzilov, Mitchell E. Skinner, Yuri R. Bendaña, Lars Barquist, Ian Holmes

**Affiliations:** 1 Biophysics Graduate Group, University of California, Berkeley, California, United States of America; 2 Department of Bioengineering, University of California, Berkeley, California, United States of America; University of Oxford, United Kingdom

## Abstract

We performed benchmarks of phylogenetic grammar-based ncRNA gene prediction, experimenting with eight different models of structural evolution and two different programs for genome alignment. We evaluated our models using alignments of twelve *Drosophila* genomes. We find that ncRNA prediction performance can vary greatly between different gene predictors and subfamilies of ncRNA gene. Our estimates for false positive rates are based on simulations which preserve local islands of conservation; using these simulations, we predict a higher rate of false positives than previous computational ncRNA screens have reported. Using one of the tested prediction grammars, we provide an updated set of ncRNA predictions for *D. melanogaster* and compare them to previously-published predictions and experimental data. Many of our predictions show correlations with protein-coding genes. We found significant depletion of intergenic predictions near the 3′ end of coding regions and furthermore depletion of predictions in the first intron of protein-coding genes. Some of our predictions are colocated with larger putative unannotated genes: for example, 17 of our predictions showing homology to the RFAM family snoR28 appear in a tandem array on the X chromosome; the 4.5 Kbp spanned by the predicted tandem array is contained within a FlyBase-annotated cDNA.

## Introduction

The number of non-coding RNAs (ncRNAs) in eukaryotic genomes is one of the pressing open questions of genomics. The upper bound on this number is believed to be in the tens of thousands [1]. The biological significance of ncRNA is supported by several recently-discovered classes of RNA that have function at the transcript (as opposed to protein) level. These include independently-transcribed gene families such as microRNAs (miRNAs) [2], [3], small nucleolar RNAs (snoRNAs) [4], and piwiRNAs [5], as well as functional RNA elements in protein-coding genes such as riboswitches [6], zipcodes [7] and splicing regulators [8]. Microarray transcriptome surveys [9], as well as whole-genome bioinformatics screens, turn up thousands of candidate ncRNAs [10]–[13].

One of the comparative-genomics approaches used to find non-coding RNAs involves stochastic context-free grammars (SCFGs) [14], [15]. In particular, phylogenetic SCFGs or “phylo-grammars” have been used to scan multiple genome alignments for ncRNAs [16]. Phylo-grammars are powerful, parameter-rich models of the spatial and temporal structure of evolving genomic features. As well as for *de novo* ncRNA annotation, they have been used to detect protein-coding genes [17], [18], conserved regions [19] and fast-evolving ones [20]. They simultaneously model several aspects of features under consideration, including the sequential organization (e.g. nesting of base-pairs and length distributions of stems and loops) and base composition of genomic sequence, the rates of point substitution at individual sites and covariant substitution at functionally coherent groups of sites (such as base-pairs or codons), and the underlying phylogeny, including both branch lengths and tree topology. A particular strength of the phylo-grammar framework is the ease with which it is (theoretically) possible to refine the models, adding new components to better model target features [21] or altering the parametric structure of the substitution rate matrices, a common practice when training data are sparse [22]–[24].

Although the framework is flexible, implementing a phylo-grammar is difficult and effectively parameterizing one is even harder. Consequently, while there have been recent comparative studies of non-phylogenetic SCFGs for secondary structure prediction [21], there have been no such comparative studies of phylo-SCFGs for gene detection, despite two gene-predicting phylo-SCFGs having been published [16], [25].

We previously described a general-purpose software package for prototyping, parameter-fitting and alignment annotation using phylo-grammars [26]. This program, **xrate**, allows the grammar structure to be specified in a configuration file; the parameters can then be automatically estimated from training data and the parameterized phylo-grammar used to annotate new alignments. This program implements a wide variety of models and can be used for measurement of evolutionary rates, or prediction of RNA (or protein) secondary structure.

In this paper, we report the first use of **xrate** for ncRNA gene prediction. Estimating false-positive rates using simulated data, we evaluated our methods on the twelve genome sequences in the *Drosophila* species clade [27], [28]. There are 942 annotated ncRNAs (including both independent transcriptional units and regulatory elements within genes) in *D. melanogaster* (FlyBase release 5.4) and several whole-genome transcriptomics studies [9], [29].

Our method involves breaking a multi-genome alignment into 300-nucleotide windows (with 100-nucleotides overlap between adjacent windows), scanning each window with a phylo-grammar to find the highest-scoring potential structured RNA within each window and selecting predictions above a certain score cutoff. Starting with the **PFOLD** phylo-grammar of [30], we test several refinements to the method: new parameter-fitting algorithms, more biophysically-realistic RNA structure models, better null models for neutrally-evolving intergenic sequence, variations in insertion and deletion rates and two different genome alignment algorithms.

Using one of the grammars, we scan a multiple alignment of twelve *Drosophila* genomes for novel ncRNAs. As well as reproducing many of the predictions of earlier bioinformatics screens in *Drosophila* [11], [13], [28], our screen predicts numerous novel structured RNAs, lending support to the hypothesis that eukaryotic genomes are dense with ncRNAs. However, the simulation procedure that we use (which includes locally conserved regions that are *not* ncRNAs) suggests that false positive rates for ncRNA prediction are higher than previously reported. We find many correlations between our predictions and coding regions in *D. melanogaster*, including depleted numbers of predicted intergenic ncRNAs near the 3′ end of coding regions as well as fewer predictions in the first intron of known protein-coding genes than expected by chance. Our methods point the way to further evidence-based evaluations of whole-genome bioinformatics screens.

## Results

All of our results may be accessed at the following URL: http://biowiki.org/TwelveFlyScreenPredictions

### Design of ncRNA gene model

We tested several models for prediction of structured ncRNAs. Each model contained two “submodels”: a *ncRNA model* to model the structural evolution of the ncRNA, and a *null model* to model the neutral evolution of the remaining sequence in the window.

We evaluated the performance of ncRNA gene models using test datasets of true positives constructed by extracting sub-alignments of annotated ncRNAs in FlyBase Release 5.4 of the *D. melanogaster* genome from multiple alignments of twelve *Drosophila* genomes (*melanogaster*, *pseudoobscura*, *sechellia*, *simulans*, *yakuba*, *erecta*, *ananassae*, *persimilis*, *willistoni*, *mojavensis*, *virilis* and *grimshawi*; see Methods for details) [27]. In contrast to thermodynamic methods, which explicitly model RNA structures including loop length and base-stacking effects, phylo-grammar-based gene models primarily score candidate structured sequence based on the statistical evolutionary signal that the structure leaves in the multiple alignment, rather than the energetics of the structure itself. Our model evaluation procedure is primarily a testbed for selecting an appropriate substitution model for stems, loops and neutrally-evolving sequence (see “Patterns of nucleotide substitution in non-coding RNA”). To help reduce bias, we created four different test sets, one of (highly-conserved) tRNAs, one of miRNAs, one of snRNAs, snoRNAs and other RNAs, and one of all non-ribosomal RNAs. We excluded rRNAs from our analysis because they are unaligned.

In each case, the ncRNA model was derived from the **PFOLD** model [30], a lightweight grammar known to perform well at single-sequence structure prediction [21]. This grammar (and all the derivatives that we tested) are capable of modeling the salient features of ncRNA secondary structure (including hairpins, bulges, interior loops, and multi-branch loops). The **PFOLD** rate parameters were estimated approximately, by counting mutations in the Bayreuth tRNA database [31] and the European large subunit rRNA database [32]. The counting technique used by Knudsen & Hein is likely to under-count certain mutations, and is an approximation to a true Maximum Likelihood (ML) estimate. Our first derivative model used the same grammar structure as **PFOLD**, but with rate parameters independently re-estimated from similar alignment data, using **xrate**'s EM algorithm, which gives a closer approximation to ML.

Several of our derivative ncRNA models include more detailed modeling of RNA structure. The ClosingBp grammar (which we eventually chose for our whole-genome screen) takes account of the substitution patterns of the loop-closing base-pair at the end of a stem, which frequently differ from the patterns observed within the stems [33]. The SymmetricStemGaps, NoStemGaps, GapLinks and GapSub grammars included various models for indel events in stem and loop regions. These ranged from allowing indels in base-paired regions only if both bases in a pair were deleted (SymmetricStemGaps), to prohibiting indels entirely in base-paired regions (NoStemGaps), to explicit probabilistic models for gaps, either as a birth-death process (GapLinks) or a substitution-based process (GapSub).

In all cases, the null model was trained on a random 1% of the **PECAN** *Drosophila* alignments. In all but one case, the null model was a single-nucleotide “point substitution” model that was reversible and strand-symmetric (but otherwise fully general). The exception was the Dinuc model, where we allowed the substitution rates in the null model to be “context-dependent” (so the substitution patterns at a given site depend on the neighboring sequence). Previous studies of codon-emitting phylogenetic Hidden Markov Models for protein-coding gene prediction have shown that such phylo-HMMs tend to over-predict exons unless context-dependent substitution effects are included in the null model [18]. It is hypothesized that this is due to the implicit inclusion of neighbor-dependent substitution effects in the codon evolution model; unless those effects are included in the null model too, the codon model has an “unfair” advantage.

**Figure 1:**
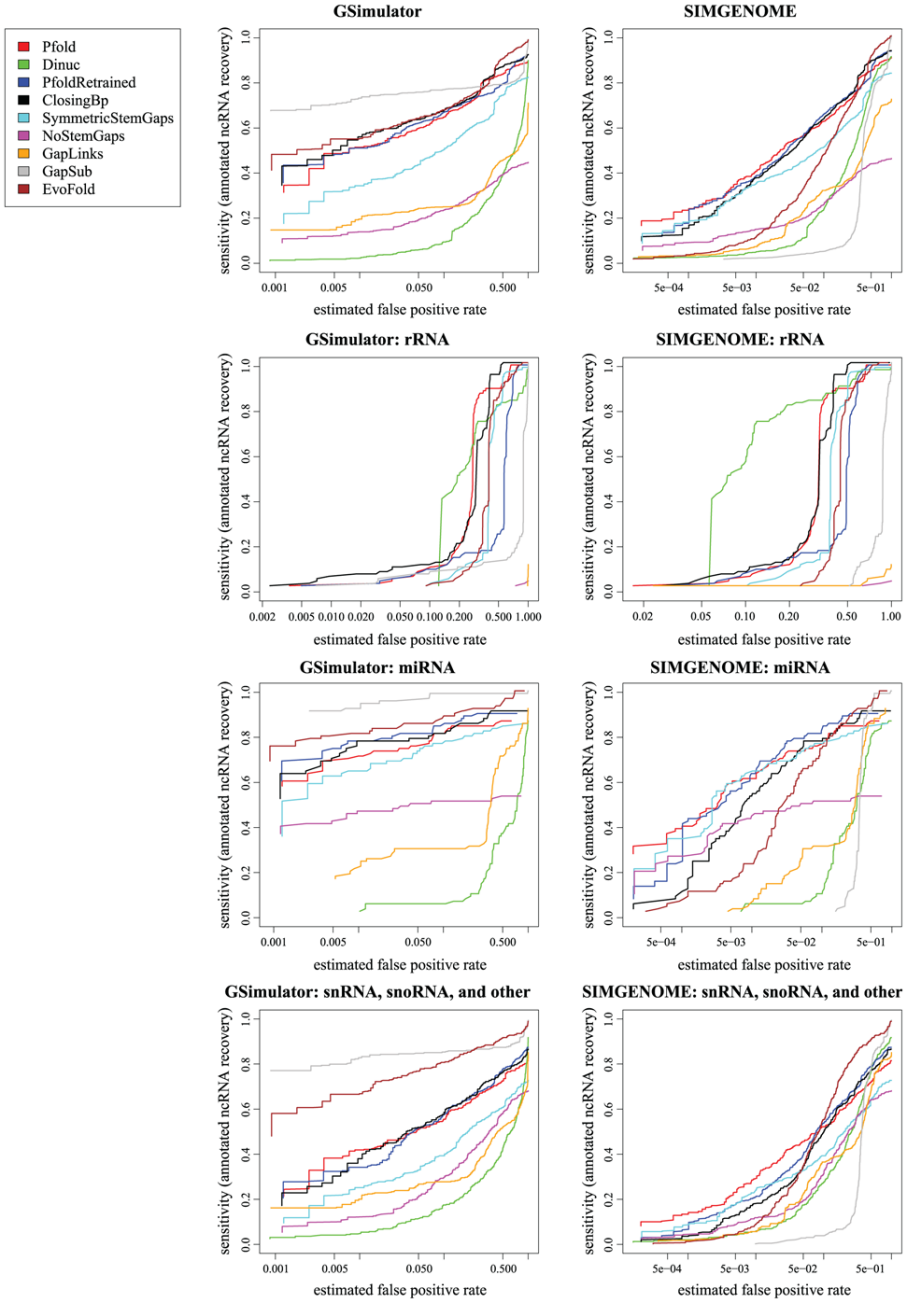
ROC curves comparing ncRNA gene prediction performance on various subsets of *D. melanogaster* ncRNAs. The ROC curves on the left used simulated data generated by gsimulator, which models neutrally evolving DNA (i.e., loosely speaking, intergenic regions). The ROC curves on the right used simulated data generated by simgenome, which additionally includes conserved signals such as protein-coding exons (i.e. it models both intergenic and gene regions). Both simulated datasets were re-aligned with PECAN prior to gene-prediction. Each row represents a different subset of true *D. melanogaster* ncRNAs: the top row includes all ncRNAs, the second row rRNA only, the third row miRNA only, and the bottom row includes snRNAs, snoRNAs and other “small” families (excluding tRNA and rRNA). We tested several prediction grammars including “Pfold”, based on the original PFOLD grammar [30]; “PfoldRetrained”, a version of PFOLD reparameterized from the mix80 dataset [38]; “Dinuc”, a derivative of PFOLD with a dinucleotide null model; “ClosingBp”, a derivative of PFOLD that explicitly models the closing basepair statistics of loops; “SymmetricStemGaps”, a derivative of PFOLD that excludes deletions of only one half of a basepair; “NoStemGaps”, an even stricter derivative of PFOLD that excludes gaps in stems altogether; “GapLinks”, a PFOLD-derivative that approximately models gaps as a birth-death process; “GapSub”, a PFOLD-derivative that approximately models gaps as a substitution process; and “EvoFold”, the grammar used by the program EvoFold [10]. The horizontal axis (false positive rate) is plotted logarithmically, so as to reveal the behavior in the low-false-positive regime, which is primariy of interest (the left-hand side of the plots). Note that these screens were performed on *aligned* genome data, and in particular, note that not all of the genome is contained within such alignments. Our procedure can only discover ncRNAs that are contained within one of the aligned regions. Since some of the *D. melanogaster* ncRNAs are not contained within the PECAN alignments, these ncRNAs are never discovered; hence, the sensitivity never reaches 1 in these curves (so they are non-standard ROC curves in that sense).

Figure 1 shows ROC curves for the grammars we tested, using various subsets of the annotated *D. melanogaster* ncRNAs. Detailed specifications for the grammars are as follows:

1. Pfold: original **PFOLD** grammar, including the original rate parameters; single-nucleotide null model of intergenic sequence (context-independent).
2. Dinuc: original **PFOLD** grammar, including the original rate parameters; dinucleotide null model (nearest-neighbor context dependence).
3. PfoldRetrained: original **PFOLD** grammar, but with rates re-estimated from the **mix80** dataset using **xrate**'s EM algorithm. Single-nucleotide null model.
4. ClosingBp: **mix80**-trained rates; closing base-pair of loops can optionally use a separate substitution rate matrix. Single-nucleotide null model.
5. SymmetricStemGaps: original **PFOLD** grammar, including the original rate parameters; gaps in stems permitted only if both sites of a base-pair are gapped. Single-nucleotide null model.
6. NoStemGaps: original **PFOLD** grammar, including the original rate parameters; no gaps allowed in stems. Single-nucleotide null model.
7. GapLinks: **mix80**-trained rates; approximate birth-death or “links” model [34] for runs of gaps in stems, loops and intergenic sequence. Single-nucleotide null model.
8. GapSub: **mix80**-trained rates; gaps are treated as a fifth character in both ncRNA and intergenic sequence [35]. Single-nucleotide null model.
9. EvoFold: the ncRNA grammar used by the program **EvoFold** [10]; single-nucleotide null model of intergenic sequence (context-independent).

Several of these grammars model features which, to our knowledge, have not previously been used for *de novo* ncRNA annotation, including closing-base-pair statistics, strict stem conservation and explicit models of the insertion and deletion process.

We used two different methods for generating simulated decoy alignments in order to estimate the false positive rate. These methods were **gsimulator**, which essentially generates intergenic DNA, and **simgenome**, which generates signals like exons as well as “neutral” intergenic sequence [36]. If we knew the correct annotation of every protein-coding exon, and we were only looking for ncRNAs in known intergenic regions, then **gsimulator** would be the most appropriate tool; if, on the other hand, we had zero information about protein-coding exons, and were predicting genes blindly in an unannotated genome, then **simgenome** would be more appropriate. The reality is somewhere in between; for *D. melanogaster*, where most (but not all) exons are now believed to be known with confidence, it is probably closer to **gsimulator**.

Due to the large number of false positives in these screens, we are interested primarily in the sensitivity of the grammars when the false positive rate is lowest, i.e. the left-hand side of the plot. The x-axis of the plots is shown logarithmically in order to better highlight the performance in this regime.

In general, the relative performance of the different grammars varied wildly across different ncRNA subfamilies and different methods for generating null/decoy datasets. The **PFOLD** grammar in particular performed relatively weakly when the null dataset was generated by **gsimulator** (which has low GC content and a low degree of conservation), but was the strongest when using a **simgenome**-generated dataset (wherein the GC content is closer to uniform and the substitution rate more heterogeneous, thanks to conserved information-rich regions such as exons). Conversely, the EvoFold and ClosingBp grammars performed well on the **gsimulator** test, but poorly on the **simgenome** test.

The ClosingBp grammar, which was designed to model a phenomenon specifically observed in rRNA [33], generally performed better on the rRNA benchmark than on the others. The Dinuc grammar, which differs from the **PFOLD** grammar only in its null model, also performed better on rRNA.

Of the four gap models we tried, only the substitution-based model (GapSub) seems to yield a significant improvement; this may be because the birth-death model which we tried (GapLinks) was actually a single-event approximation to a true birth-death process, and so is under-normalized probabilistically. The shape of the ROC curves for the gap models may suggest that the performance could benefit from a null-model that explicitly modeled regions with no or few gaps.

We found that the Dinuc grammar, with a strand-symmetric dinucleotide model of intergenic sequence, underperformed on our test datasets, with the exception of rRNA (Figure 1). A dinucleotide model of sequence can capture local correlations, whereas our ncRNA gene model captures only long-distance correlations due to secondary structure. We hypothesize that a dinucleotide model of intergenic is “too good” for our current gene model: in situations where the structural-conservation signal is weak, whether due to little base-pairing or poor alignments, local correlations may contribute more to the sequence likelihood than secondary structure. A dinucleotide model of intergenic sequence may be well-suited to a more elaborate ncRNA model which captures local correlations such as base-pair stacking effects. This may be a general rule for detecting conserved elements: the conserved-element model should be capable of modeling all correlations, local or long-distance, represented in the null model.

**Figure 2:**
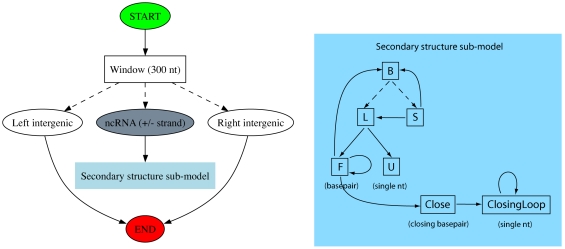
Design of the ClosingBp grammar. The left figure gives an overview of our approach and the right figure a detailed picture of the secondary structure submodel for structured RNA. The state labeled “ncRNA (+/− strand)” chooses the strand of the structured element. Solid arrows are transitions from a single state to another state and dotted arrows are multifurcations (transitions from one state to a set of states). In the right panel, emit states have the symbol being emitted labeled in parentheses under the state.

We chose the ClosingBp grammar for our whole-genome screen, it being a novel **PFOLD** derivative which appeared to give good performance in the gsimulator test (i.e. on intergenic DNA). The basic elements of this grammar are illustrated in Figure 2.

### Patterns of nucleotide substitution in non-coding RNA

**Figure 3:**
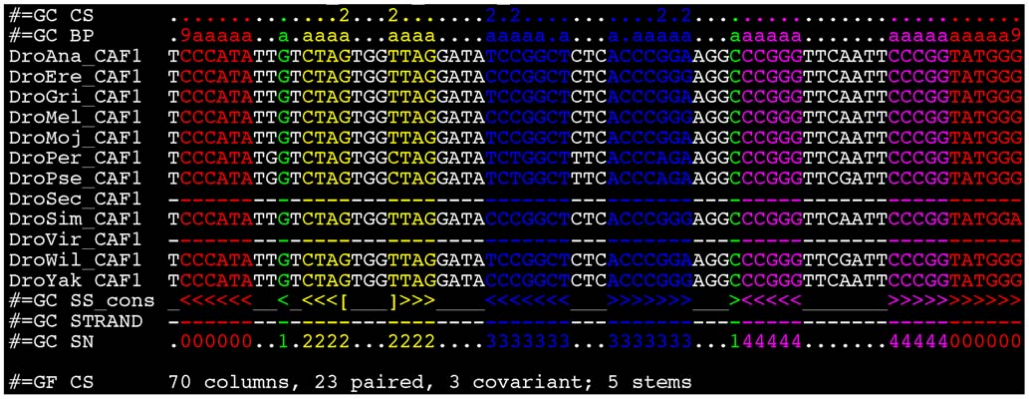
Recovery of a tRNA (FlyBase gene identifier FBgn:0050220) on chromosome 2R. We recover the four stems of the classic cloverleaf structure, as well as a spurious single base-pair annotated as stem 1 (green). The 5′ boundary is exactly recovered and the 3′ boundary is 2 nt shorter than the FlyBase annotation. Note that stems 2 and 3 (yellow and blue) have, respectively, one and two compensatory mutations. If a base pair exhibits compensatory mutations, the “CS” row shows the count of distinct canonical base-pairs in the columns. The “BP” column shows how many sequences contain a canonical base pair in the consensus structure (“a” = 10). The “SS_cons” row indicates the ML consensus secondary structure predicted by our model; colors of nucleotides and numbers in the “SN” row indicate the stems of this predicted structure. Figure produced with colorstock, described by [51]. The alignment is in the Stockholm file format used by RFAM [37].

Compensatory substitutions in ncRNA stems, where paired bases can be seen as evolving together as a coherent unit (just as codons evolve as coherent units in protein-coding genes), are a classic signal of structural conservation. For example, Figure 3 shows a tRNA exhibiting compensatory substitutions at 3 sites. The substitution rates of these paired mutations describe the constrained molecular evolution of structured RNAs and as such must be chosen carefully to maximize the predictive power of our model.

We used the EM algorithm to estimate ncRNA substitution rates from two datasets: (1) a subset of multiple alignments from release 7 of RFAM [37] whose annotated secondary structure was derived from a published source; (2) a set of pairwise alignments derived from the **mix80** dataset used to parameterize the **CONSAN** program [38], which in turn was derived from the European Ribosomal RNA database. In each case, we estimated a phylogenetic tree for the dataset using the Jukes-Cantor model, then used this tree in estimating the rates. We did not enforce that the substitution rate matrices be normalized to one expected substitution per unit of time (as is common in some molecular evolution analysis), since we wanted to account for the fact that stem regions evolve more slowly than loop or intergenic regions.

**Figure 4:**
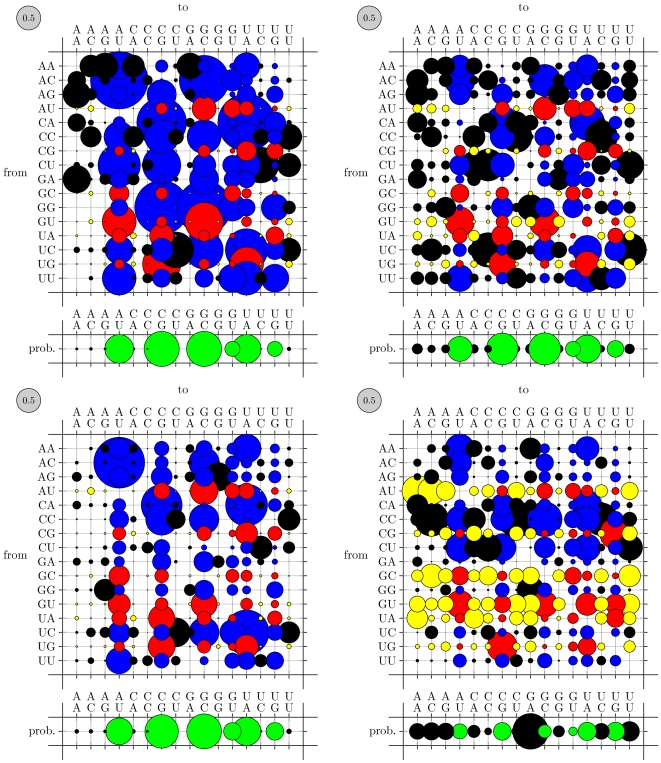
Substitution rate matrices for co-evolving base-pairs. The area of each bubble is proportional to the corresponding rate (the gray bubble in the upper-left of each plot shows the scale: its area corresponds to 0.5 substitutions per unit of time). The color of a bubble indicates whether the source and destination base-pairs are canonically paired. (Red, Yellow) circles show substitutions from canonical to (canonical, non-canonical) base-pairs; (Blue, Black) show substitutions from not paired to (canonical, non-canonical). The area of the bubbles in the row beneath each plot indicates the equilibrium distribution of the mutation process (canonical base pairs are green, non-canonical are black). The RFAM-trained rates (upper right) show higher rates of mutations away from canonical pairings than do the mix80-trained rates (lower left) or the original PFOLD rates (upper left). In the closing base-pair of stems (lower right) one can observe a bias towards G-A base-pairs in the equilibrium distribution as well as high rates of mutations away from canonical pairings (yellow bubbles). See “Patterns of nucleotide substitution in non-coding RNA” for further details.

Figure 4 compares these re-estimated base-pair substitution rates to those of **PFOLD**, on which our grammar models were originally based. The most notable difference is that both datasets exhibit significantly slower rates than **PFOLD**'s. More subtly, the RFAM-trained rate matrix (middle) has a noisier equilibrium distribution, assigning greater weight to non-canonical base-pairs, than the **PFOLD** matrix (left). This resulted in significantly deteriorated performance at gene prediction relative to **PFOLD** (results not shown). Speculating that this may have been due to mis-annotated base-pairs in RFAM (which applies a consensus secondary structure to every sequence in an alignment), we next used the **mix80** dataset, where each sequence is individually annotated with its own structure. This dataset is also closer to the dataset of rRNAs which was used to parameterize **PFOLD** (B.Knudsen, personal communication). As can be seen from Figure 4 (right), the **mix80** dataset has a sharper split between non-canonical and canonical base-pairs, more similar to **PFOLD**'s (left).

One of our variations on the **PFOLD** model was to allow, although not require, a separate substitution model for base-pairs at the ends of stems (i.e. the closing base-pair of a loop), where a bias towards G-A and A-A base-pairs has been observed in ribosomal RNA [33]. This grammar is illustrated in Figure 2 (note that only the Close and ClosingLoop states are new; the remainder of the grammar is taken from **PFOLD**, so that **PFOLD**'s mechanism for generating loop regions — via the transition F→B — remains a viable alternative to the new states.) Figure 4 compares the matrix thus obtained (lower right) to the matrix for regular base-pairs (lower left). We observe a bias to G-A base-pairs (although no A-A bias), and furthermore see little evidence for compensatory mutations in these positions.

### Recovery of known ncRNAs

Table 1 shows our recovery rates, broken down by category, of ncRNAs annotated in FlyBase release 5.4 [39]. The results in this table are generated using our ClosingBp grammar, one of the highest-performing according to our benchmarks (see “Design of ncRNA gene model”).

**Table 1:** Recovery of annotated ncRNAs in *D. melanogaster*, where ncRNA annotations are taken from FlyBase Release 5.4.

	miRNA	tRNA	snRNA	snoRNA	RNaseP	other
Recovered	56	246	17	64	1	27
% of total	62%	84%	36%	26%	100%	31%

Results are not reported for the unaligned rRNAs.

Our method largely scores conservation of RNA secondary structure according to observed compensatory mutations within stems, and as such is most effective at picking up well-conserved ncRNAs with long hairpins or several stems. We successfully recover the majority of annotated miRNAs and transfer RNAs (tRNAs); the long hairpins of processed primary transcripts of miRNA (pre-miRNA) and four stems of tRNAs make both relatively easy for our method to detect. Many C/D box snoRNAs, in contrast, have too few base-pairs to score well under our method.

### Statistics of predicted ncRNAs

**Figure 5:**
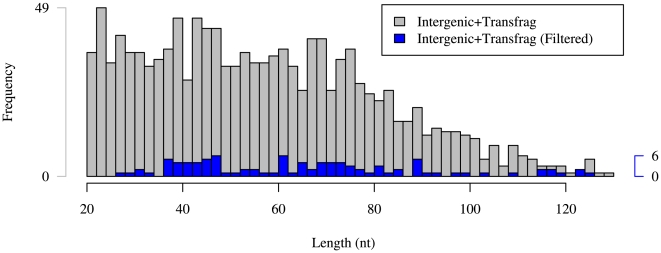
Length distribution of predictions in intergenic sequence which overlap embryonic transcriptional data. Grey denotes all Intergenic+Transfrag predictions and blue denotes Intergenic+Transfrag predictions which pass our filtering criteria. Longer predictions, with their generically longer stems, are more likely to exhibit the compensatory mutations required by our filtering criteria, thereby flattening the distribution.

Table 2 shows the chromosomal distribution of our predicted ncRNAs and Figure 5 gives the length distributions of our predictions in intergenic sequence which overlap embryonic transcriptional data before and after filtering criteria are applied. The filtered predictions are in general slightly longer than the unfiltered predictions, and their length distribution is slightly flatter.

**Table 2:** Chromosomal distribution of our predicted ncRNAs in *D. melanogaster*.

	2L	2R	3L	3R	4	X
Predictions	9,644	9,787	11,534	13,341	225	11,557
Filtered Predictions	2,846	3,001	2,953	3,716	119	2,720

Our filtering procedure to obtain high-quality predictions for experimental verification is described in detail in Methods.

Several other whole-genome screens for novel ncRNAs in *Drosophila* have recently been conducted, including computational screens for structured RNAs using the programs **RNAz** [11], [40] and **EvoFold** [10] as well as an experimental screen for miRNAs by [13]. Table 3 shows the intersection of our predictions with those reported from the **RNAz** screen. We found little overlap between our prediction sets, despite both methods using the same **PECAN** alignments as input. As reported in Table 4, we find greater overlap with the prediction set produced with **EvoFold** [28], which uses a phylo-grammar-based approach similar to ours. This is encouraging, given that **EvoFold** was run on the **MULTIZ** alignments, which use an entirely different synteny map from the **PECAN** alignment. We recovered 65 (44%) of the miRNAs predicted by the recent experimental screen [13]. We found no significant correlation between overlap with the results of **EvoFold**, **RNAz** or other [13] screens and the phylogenetic conservation (% identity) of the overlapping predictions.

**Table 3:** Comparison to **RNAz**'s results.

Category	Prediction overlap
*p*>0.5	4,163 (10%)
*p*>0.9	1,658 (10%)

**RNAz** reports 42,482 predictions at a confidence level of *p*>0.5, so we compared those predictions with our best-scoring 42,482 predictions.

**Table 4:** Comparison to **EvoFold**'s results.

Category	Prediction overlap	Total overlap
Short	1,855 (14%)	6,436 (50%)
Long	2,239 (22%)	6,225 (62%)
HighConf	96 (16%)	151 (25%)

The center column shows the recovery rate across our predictions and the right column the recovery rate across all of our annotated structures, including those which did *not* meet our discovery threshold (Methods). Our predictions in each category (Short, Long and HighConf) were filtered per **EvoFold**'s analysis and then compared with **EvoFold**'s predictions.

Taken together, these comparisons with previous approaches suggest that no single method assembles a complete catalog of ncRNAs. It is best to regard the various prediction sets as complementary. In particular, phylo-grammar-based genome screens run on different whole-genome alignments can recover distinctly different prediction sets corresponding to the different phylogenetic signals present in the input alignments.

### Finding homologues to characterized RNAs

We screened our unfiltered, non-overlapping intergenic predictions in *D. melanogaster* against the RFAM database with the *Infernal* ncRNA homology search tool [41]. 114 of these predictions showed significant homology to a RFAM family, including 2 predictions scoring as tRNAs, 22 as miRNAs, and 36 as snoRNAs. Relatively few of these predicted tRNAs, miRNAs or snoRNAs were predicted by other whole-genome screens; Table 5 gives a detailed breakdown.

**Table 5:** We used the **cmsearch** utility provided with **Infernal** to search for homology to known ncRNA families in our intergenic filtered prediction set.

	Overlap with other ncRNA gene sets:			
	Predictions	RNAz	EvoFold	[13]
tRNA	2	1		
miRNA	22	2	1	2
snoRNA	36	8		
other	54	13		

Results reported here had a bit score>16.4 (see “Screening predictions against RFAM” for details).

As suggested earlier, our predictions may be associated with introns of unannotated protein-coding genes. 19 of our predictions scoring as snoRNAs correspond to the single RFAM family snoR28, and 17 of these appear in a tandem array on the X chromosome. The 4.5 kbp spanned by the predicted tandem array is contained within a cDNA annotated in FlyBase, suggesting that our predictions lie within intronic sequence of an unannotated protein-coding gene.

### Associations with protein-coding genes

**Figure 6:**
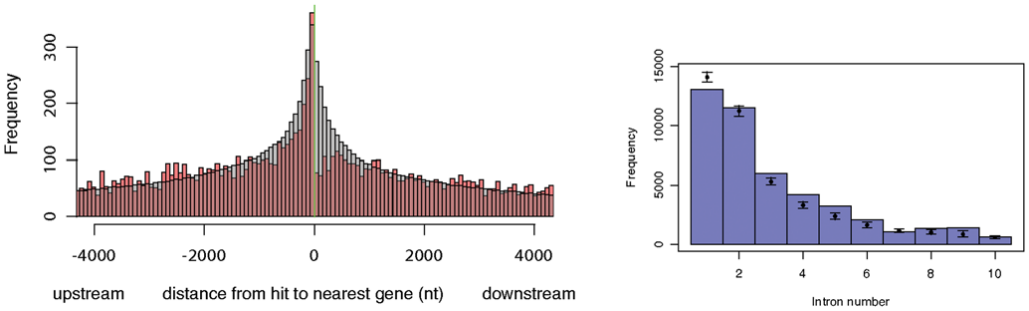
Association of the top-scoring 5% of predictions with protein-coding genes. Left: distance from intergenic predictions to the nearest protein-coding gene. “Upstream” means the prediction is upstream of the gene. The red bars show the empirically observed distribution; the grey bars show the distribution that would be expected if hits were uniformly distributed across the genome. We observe a clear depletion of intergenic predictions near the 3′ end of protein-coding genes. Right: frequencies of predictions enclosed completely by introns, separated by intron number (i.e. the position of the intron in the ordered list of introns associated with the parent gene). The blue bars show the empirically observed counts; the error bars show 99% bounds for a uniform random distribution of bases across all chromosomes (excluding bases outside introns). Introns shared by multiple transcripts were counted multiple times. There is a depletion of predictions in the first intron.

As reported in Figure 6, we found a small (but significant) depletion of predictions near the 3′ end of protein-coding genes as well as depletion of predictions in the first intron. The depletion of 3′ predictions might conceivably be due to unannotated exons. Depletion of predictions in the first intron is harder to explain; it is possible that other conserved signals in the intron either exclude real ncRNAs from these locations, or result in fewer false positives under our prediction screen.

**Figure 7:**
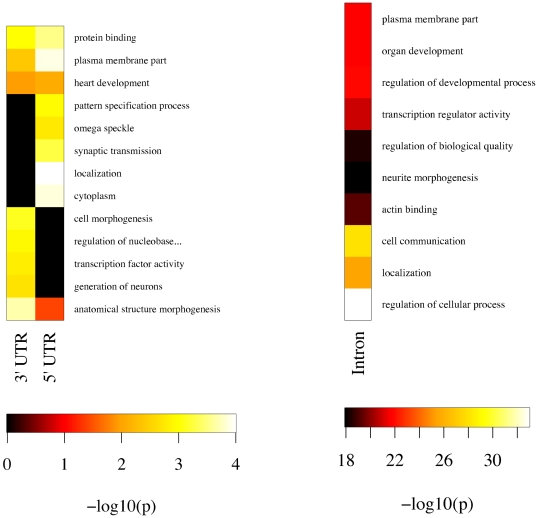
Gene Ontology term enrichment for protein-coding genes with (filtered) predicted structured RNAs in 3′ and 5′ UTRs and introns. The heat map color scale indicates statistical significance (white is most significant). We selected the forty most-significant terms for UTRs and introns and then removed terms if a descendant in the GO was also present. ”Regulation of nucleobase, nucleoside, nucleotide and nucleic acid metabolic process” is truncated in the UTR figure (left) for readability.

As a first step towards functional characterization of protein-coding genes with predicted structurally-conserved elements in their 3′ and 5′ untranslated regions (UTRs) and introns, we identified enriched Gene Ontology (GO) terms with GO::TermFinder [42]. Figure 7 indicates potential biological functions for the structured elements we identify. Many of these terms suggest functional roles in localization processes and transcriptional regulation, including “pattern specification process,” “localization,” “protein binding” and “transcription factor activity” for UTRs and “localization,” “actin binding” and “transcription regulator activity” for introns, suggesting that these predicted structured elements may play regulatory roles. A recent survey of 3,370 genes in *D. melanogaster* found that 71% exhibited subcellular localization of the corresponding mRNA in the first 4 hours (stages 1–9) of embryogenesis [29]. In the context of this result, our predictions in 3′ and 5′ UTRs are of particular interest. The localization signals for the vast majority of the mRNAs studied by [29] are completely uncharacterized, and many of our predicted structurally-conserved elements in 3′ and 5′ UTRs and introns may represent novel signal elements for subcellular localization.

## Methods

### Sequence and alignment data

We used alignments of twelve *Drosophila* genomes (*melanogaster*, *pseudoobscura*, *sechellia*, *simulans*, *yakuba*, *erecta*, *ananassae*, *persimilis*, *willistoni*, *mojavensis*, *virilis* and *grimshawi*) which were produced by the *Drosophila* Twelve Genomes Consortium [27]. These alignments used the Comparative Assembly Freeze 1 (CAF1) sequence data, which includes Release 4 of the *melanogaster* genome and release 2 of the *pseudoobscura* genome. The other ten genomes were newly-sequenced [27]. Both the **MAVID** [43] and **PECAN** [44] alignments of the CAF1 data used a homology map produced with the **Mercator** program [45]. The **PECAN** and **MAVID** alignments used in our analysis can be downloaded from our results page.

Unless noted otherwise, we used annotations from FlyBase Release 5.4 of the *D. melanogaster* genome for our analysis, including recovery of annotated ncRNAs (Table 1) and analysis of predictions in UTRs of *D. melanogaster*. These annotations use the same co-ordinate scheme with respect to *D. melanogaster* (i.e. assembly) as the CAF1 alignments.

### Simulations of neutral evolution

We used simulated alignment data to guide the design of our ncRNA discovery pipeline and estimate the corresponding false-positive rate. A good synthetic dataset should reproduce empirically-observed features of actual alignments, including gap (indel) structures and local correlations between nucleotides, both of which locally deplete the information content of an alignment and can elevate false-positive rates.

We generated synthetic alignments by forward simulation of the evolutionary process with the **simgenome** program [36] followed by re-alignment with **PECAN** [44]. **simgenome** models the evolution of syntenic blocks of the genome. Genomic features, including coding and intronic sequence, locally-conserved regions, pseudogenes, and DNA transposons, are modeled with a phylo-grammar; neutrally-evolving intergenic sequence is modeled with a “transducer,” a probabilistic model which explicitly incorporates indel length distributions and the effect of local sequence context on substitution and indel rates [46]. Table 6 compares genome-wide statistics of our simulated data with those of the **PECAN** alignments of twelve *Drosophila* genomes and Table 7 the single and di-nucleotide frequencies.

**Table 6:** Genome-wide statistics of our simulated alignments of twelve *Drosophila* genomes closely match those of the true data.

Dataset	% ID	% gap	% coding	% intronic
**PECAN**	83%	89%	33%	18%
**simgenome** (realigned)	85%	83%	33%	18%
**simgenome** (original)	69%	41%	33%	18%

The average length of simulated alignments was 240K columns, in contrast to the 142K for the **PECAN** alignments; however, our windowing approach makes our method insensitive to the sizes of syntenic regions. We generated a total of 3.6M columns of alignment data. “**simgenome** (realigned)” is the simulated alignments after re-alignment with **PECAN** which we use for all subsequent analysis and refer to as simply “**simgenome**”. “**simgenome** (original)” is the simulated alignments generated by **simgenome**. Sequence identity and gap fraction were estimated from the **PECAN** alignments; coding and intronic fractions were estimated from [27].

**Table 7:** Single and di-nucleotide frequencies for our simulated data (left) closely match those in the twelve *Drosophila* genomes (right).

	A	C	G	T	A	C	G	T
	0.273	0.228	0.228	0.271	0.285	0.204	0.204	0.284
A	0.070	0.053	0.052	0.060	0.094	0.049	0.052	0.077
C	0.052	0.047	0.048	0.055	0.065	0.041	0.036	0.051
G	0.055	0.049	0.047	0.050	0.051	0.053	0.041	0.048
T	0.058	0.052	0.054	0.069	0.061	0.051	0.065	0.094

Our simulated data models heterogeneity in base composition across different genomic features such as coding and intergenic sequence, but does not model local fluctuations in base composition.

**Figure 8:**
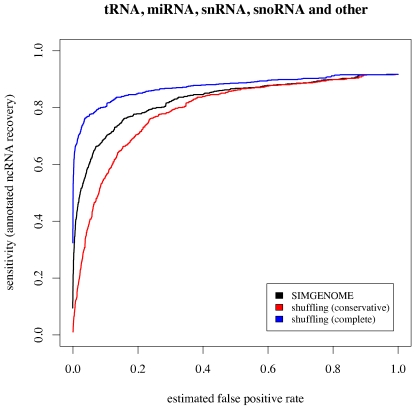
Receiver Operator Characteristic (ROC) curves for the ClosingBp grammar (see “Design of ncRNA gene model”), using our simulated data and two modes of shuffling to generate true-negative datasets. True-positive datasets were taken from the PECAN alignments of twelve *Drosophila* genomes based on all annotated non-ribosomal ncRNAs in FlyBase Release 5.4 of the *D. melanogaster* genome. False-positive estimates from a shuffling-based approach depended strongly on the amount of shuffling. We ran the shuffle-aln.pl script provided with the Vienna RNA package [52] in “conservative” and “complete” modes to create shuffled alignments of all annotated ncRNAs in *D. melanogaster*.

Previous ncRNA annotation efforts have generated datasets of negatives by shuffling actual genome alignments [40] rather than simulating the evolutionary process. Figure 8 shows a comparison of false-positive estimates generated by our simulation method with those estimated with a shuffling-based approach. We found that our false-positive estimates depended strongly on the amount of shuffling used. There is no obviously correct number of shuffles: excessive shuffling can destroy local correlations, but insufficient shuffling may leave signals of real ncRNA genes. Further complications arise from the need to preserve alignment gap statistics. Gaps and local sequence complexity are often correlated; for example, microsatellite regions are indel-prone.

**simgenome** implements both measurement and forward-simulation algorithms. That is, one can measure parameters from data, or use the measured parameters to simulate new data. Given multiple alignments as input, the program estimates evolutionary parameters directly from these training data. If a phylogenetic tree is supplied, then the program will generate a synthetic multiple alignment. This yields a dataset of negatives, or alignments with statistical properties similar to those of the original training alignments but with no true ncRNAs present.

### Annotation pipeline design

**Figure 9:**
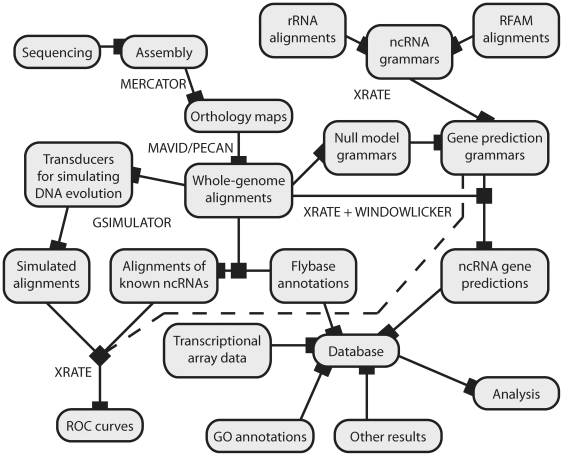
Conceptual overview of the steps in our analysis pipeline, including model parameterization (“training”); generation of simulated datasets; model evaluation (ROC curves); genome-wide prediction of conserved ncRNAs; and analysis of predictions. Rebuilding of any part of the graph is fully automated using make: Nodes represent targets and edges represent dependencies. Names of programs used in key steps (xrate, windowlicker.pl, MAVID, etc.) are shown near the relevant edges in the graph.

Several principles inform the design of our ncRNA annotation pipeline, illustrated in Figure 9. Assuming that we will re-run everything multiple times using different models or alignments on distinct species clades, we automate as much as possible using **make** and relational databases. For extensive discussion of the advantages of **make** for workflow automation, see [47]. We break the analysis into a series of discrete steps, explicitly identifying dependencies using Makefile rules, in order to easily run on new data such as different alignments or genomes from other clades. We use the **xrate** phylo-grammar engine wherever possible (for example, resolution of overlapping gene predictions on opposite strands follows automatically if a strand-symmetric grammar is used). Results and post-prediction analyses are stored in a relational database.

We divided the input multiple alignments into overlapping windows of 300 nt with a step size of 100 nt. For each overlapping window, we used **xrate** to re-estimate the branch lengths of a phylogenetic tree with the EM algorithm, and then scanned the window for conserved RNA secondary structure.

Detailed instructions for running the annotation pipeline can be found at http://biowiki.org/TwelveFlyRocCurveEstimation

### Phylo-grammar design

We chose a general-purpose approach to designing phylo-grammars in order to conduct a broad screen for signs of structural conservation without reference to particular sequence or structural motifs. While both sensitivity and specificity can be increased with methods designed to annotate only particular well-characterized families of ncRNAs, such as **Snoscan** for snoRNA detection [48], incorporating family-specific motifs (such as the conserved C (UGAUGA) and D (CUGA) boxes in C/D box snoRNAs) is incompatible with our goal of finding all structurally-conserved elements. We seek to survey the genome for novel elements showing structural, and hence potentially functional, conservation rather than catalog members of well-characterized families.

We searched each 300 nt window for the highest-scoring secondary structure element of length≤130 nt, where the score is the log-odds ratio,
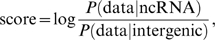
which compares the likelihood that the alignment data represents a ncRNA gene to the likelihood that it is intergenic sequence. We calculated the likelihood that the data represents a ncRNA gene by summing over all possible structures,
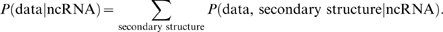



We summed over possible structures in order to classify ncRNAs in a manner agnostic to their true secondary structure. This summation over possible structures was particularly important for our ClosingBp grammar, which is structurally ambiguous [38]: Closing base-pairs of stems can evolve under either a regular base-pair model or a special substitution model estimated from the closing base-pairs of ribosomal RNA (see “Patterns of nucleotide substitution in non-coding RNA”).

### ROC curve preparation

The ROC curves were generated as follows. Whole-genome alignments and *D. melanogaster* ncRNA annotations in FlyBase release 5.4 [39] were used to estimate sensitivity, defined as
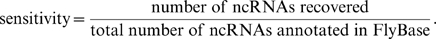



Simulated data (see “Simulations of neutral evolution”) was used to estimate the proportion of false positives (and thus the specificity) as
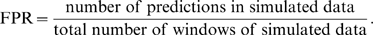



Both sensitivity and specificity are parametric functions of the score cutoff used by our discovery procedure, thereby allowing us to generate ROC curves.

We provide detailed instructions, including command-line instructions for programs, for how to duplicate our ROC analysis at http://biowiki.org/TwelveFlyRocCurveEstimation

### Filtering criteria

Because our phylo-grammar-based approach treats gaps in the alignment as missing data, our prediction method can predict RNA structures in an alignment with little or no *D. melanogaster* sequence if the other *Drosophila* genomes in the alignment exhibit signals of structural conservation. Because we are primarily interested in predictions in *D. melanogaster*, we filter out such “predictions” as described below.

Furthermore, our windowing approach gives rise to overlapping predictions. Unless specified otherwise, if predictions overlapped by more than 80%, then we retained the highest-scoring prediction and discarded the other(s).

In order to obtain a high-quality set of predictions for subsequent experimental verification, our prediction set was further reduced by applying the following stringent filters (similar to the “HighConf” filters used by **EvoFold** for [28]) to the maximum-likelihood conserved structured predicted by our model. Conserved structures were required to include at least ten base-paired columns, at least two of which had to display compensatory mutations (a compensatory mutation means a substitution at one or both sites of a base-pair such that the canonical base-pairing is preserved: for example, an A-U base-pair aligned with a G-C). Alignment segments predicted to contain conserved RNA secondary structure were discarded unless they contained at least 20 bases of *D. melanogaster* sequence and sequence from at least four other species with gaps in no more than 7.5% of predicted base-pairs.

Finally, when looking for novel ncRNA genes (as opposed to regulatory elements that might be located within protein-coding genes), we excluded any predictions that overlapped with previously annotated genes (protein-coding or non-coding), pseudogenes or transposons in FlyBase release 5.4 [39]. We further honed our prediction set by requiring overlap with transcriptional fragments identified during the first twenty-four hours of *Drosophila* development using Affymetrix tiling arrays [9], thereby obtaining the “Intergenic+Transfrag (Filtered)” prediction set referenced in the main paper.

### Screening predictions against RFAM

We extracted the *D. melanogaster* sequence for all intergenic predictions, including flanking sequence up to a total length of 100 nt. To avoid overcounting, we looked only at the 854 completely non-overlapping predictions (compared with the 885 referenced in the main paper). We then used the **Infernal** v0.81 utility **cmsearch** with all RFAM 8.1 covariance models to perform a homology search on our prediction set. RFAM and **Infernal** are available from http://rfam.janelia.org/.

The **Infernal** manual suggests a rough prediction significance cutoff on the reported bit score of log_2_ (2⋅length), where length is the length of the target sequence. The total length of the query set of 854 non-overlapping predictions is 85,526 nt, leading us to chose a cutoff of log_2_(85, 526) s 16.4 bits. When a prediction scored highly under more than one covariance model, we selected the highest-scoring model.

## Discussion

We predict approximately 1,500 novel structured RNAs in intergenic regions which overlap embryonic transcriptional fragments, as well as 3,000 in 3′ and 5′ UTRs of protein-coding genes. Of these, 100 of the intergenic predictions and 800 of the 3′ and 5′ UTR predictions show very high conservation of both sequence and structure, indicating likely functional relevance. RFAM screens against our results include 22 new miRNAs and 36 new snoRNAs. Of the snoRNAs, 19 correspond to the RFAM family snoR28, and 17 of these appear in a tandem array within an unannotated protein-coding gene.

Our approach to ncRNA discovery is distinguished from prior work by our robust evaluation of annotation models as well as a novel procedure for false-positive estimation. Our **xrate** program exposes the design of the prediction grammar in a configuration file, allowing us to easily test many different predictions models to identify their relative strengths. Combined with automation of our entire workflow, this enabled us to evaluate a wider range of prediction algorithms than previously (as well as two distinct whole-genome alignment programs; see Text S1). While this paper was in preparation, two other works discussing null models in ncRNA prediction appeared in the literature [49], [50].

Different classes of ncRNAs exhibit different patterns of molecular evolution, making the comparative model evaluation which we have described crucial to designing an effective whole-genome screen. For example, explicitly modeling the substitutions at the closing base-pairs of stems increased our recovery of tRNAs by 10%, but decreased our recovery of other ncRNAs.

As discussed throughout this work, our methodology is inherently alignment-sensitive and simply cannot detect structural conservation if the input sequence is mis-aligned. This observation, combined with the low overlap between the **RNAz**, **EvoFold**, and [13] screens, suggests that we have probably missed many real ncRNAs. [12] have recently presented a methodology for *de novo* ncRNA annotation which relies on an input multiple alignment only for homology detection, and so is capable of detecting conserved structure even in the presence of local mis-alignment. Such an approach provides a promising direction for ncRNA annotation.

At the most basic level, we are interested in investigating which features of genomic data, both in structurally-conserved and neutrally-evolving sequence, are important for *de novo* ncRNA gene annotation. The thorough approach to model training, comparative model evaluation and false-positive estimation which we have described here will allow us to predict novel genomic features with increasing precision and confidence.

## Supporting Information

Text S1 (0.33 MB PDF)
