# Supplementary material for: Evolutionary Modeling and Prediction of Non-Coding RNAs in Drosophila
Source: PLoS One. 2009 Aug 11;4(8):e6478. doi: 10.1371/journal.pone.0006478 (PMC2721679; doi:10.1371/journal.pone.0006478)
Supplement: Text S1 — (0.33 MB PDF) [file pone.0006478.s001.pdf]

Evolutionary Modeling and Prediction of Non-Coding RNAs in *Drosophila*: Text S1

Robert K. Bradley[1], Andrew V. Uzilov[2], Mitchell E. Skinner[2], Yuri R. Bendaña[2], Lars Barquist[2], Ian Holmes[1,2,*]

1 Biophysics Graduate Group, University of California, Berkeley, CA, USA

2 Department of Bioengineering, University of California, Berkeley, CA, USA

∗ E-mail: Corresponding ihh@berkeley.edu

Comparison of alignment programs

Non-coding RNAs are commonly imperfectly aligned by whole-genome alignment programs [1], limiting the possible sensitivity of alignment-sensitive methods. We tested two alignment programs, `MAVID` and `PECAN`, as inputs to our screen. Both use pairwise Hidden Markov Models (Pair HMMs) to iteratively construct a multiple sequence alignment. `MAVID` [2] performs a progressive alignment, iteratively aligning sibling species while traversing a guide tree, whereas `PECAN` [3] performs consistency alignment, using posterior probabilities to maximize the expected alignment accuracy. Several studies have suggested that consistency alignment is the more accurate technique [4–6]. We sought empirical evidence for its efficacy for phylo-grammar-based ncRNA detection.

Figure 1 shows ROC curves for the Pfold and ClosingBp grammars on the `MAVID` and `PECAN` alignments. While the overall shape of the ROC curves is similar, our method is clearly more effective on the `PECAN` alignments, suggesting that consistency checking is an effective technique for improving alignments of genomic data. Signals of structural conservation are easily destroyed by small local mis-alignments which consistency checking can help to resolve.

Further integration of alignment and annotation is desirable [7]. As discussed below, misalignment can increase false-positive as well as false-negative rates of *de novo* annotation.

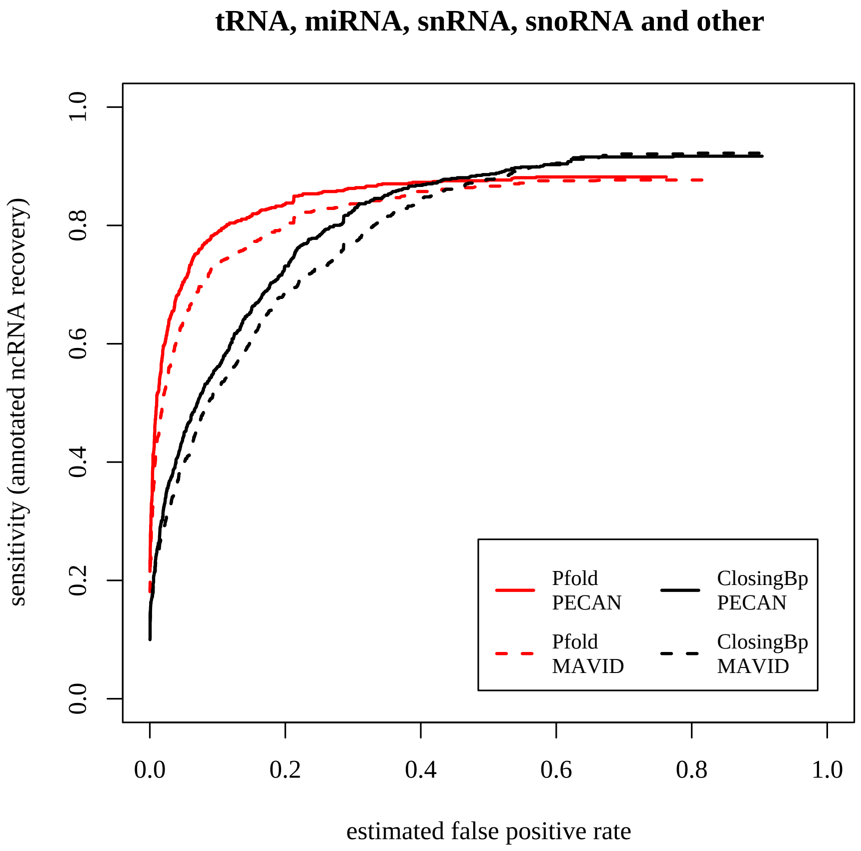

Figure 1. ROC curves for the Pfold and ClosingBp grammars on both the `MAVID` and `PECAN` alignments of *Drosophila* genomes. Simulated data was generated with `simgenome` and *not* realigned. Our ncRNA detection power is greater on the `PECAN` alignments.

Alignment error and false-positive estimation

Local mis-alignment limits the possible sensitivity of *de novo* annotation strategies which take fixed alignments as input. Perhaps surprisingly, mis-alignment can increase false-positive rates as well. Alignment programs are generally designed to maximize sensitivity, even at the price of introducing a spurious homology signal [6]. Furthermore, popular whole-genome alignment programs such as `MAVID` and `PECAN` are heuristic estimators of insertion and deletion histories and as such are unable to reliably reconstruct overlapping and nested indel events.

In order to test the effect of alignment error on our false-positive estimations, we broke the simulated data into segments of length 50,000 nt, similar to what is produced by orthology mapping programs such as `Mercator` [8], and re-aligned each segment using `PECAN`. Figure 3 shows ROC curves for the ClosingBp grammar on the simulated data before and after re-alignment with `PECAN`.

As Figure 3 suggests, re-alignment with `PECAN` generally did not significantly increase our estimated false-positive rates (except near the high-specificity discovery threshold which we chose, where the estimated false-positive rate was up to 3-fold higher). This was encouraging but surprising, given that `PECAN` introduced significant false homology and was unable to reconstruct many of the overlapping indel events present in the original data. `PECAN` correctly aligned 74% of the nucleotides aligned in the true (original simulated) alignment, but at the price of low positive predictive value (PPV): 46% of the nucleotides aligned by `PECAN` describe homology relations which are not present in the true alignment.

Our most sophisticated genome simulator allows for many overlapping and nested indel events within intergenic regions, and the indel structure of the re-alignment produced by `PECAN` was rather different from that present in the original data. We examined the evolutionary gap structure of alignment data by looking at the distribution of overlapping and nested indel events.

Overlapping indel events introduce conditional dependencies between "blocks" of the alignment, where we divide an alignment into blocks by partitioning it into the (adjacent, non-overlapping) sub-alignments defined by all gap boundaries. We say that two adjacent blocks

are conditionally dependent if an insertion or deletion event crosses the boundary between the two blocks. The number of sequential conditionally-dependent blocks indicates the number of overlapping or nested indel events; for example, three overlapping indel events give rise to three sequential conditionally-dependent blocks [9, 10].

Figure 2 shows a plot of the distribution of the number of sequential conditionally-dependent blocks before and after re-alignment. Prior to re-alignment, the many overlapping indels in the simulated data meant that each segment consisted of a single sequence of conditionally-dependent blocks; this was no longer the case after re-alignment. `PECAN` was unable to recover significant portions of the indel structure of the true alignment.

Although `PECAN`'s re-alignment introduced significant false homology and imperfectly reconstructed the true indel history, our estimated false-positive rate actually decreased as a consequence of re-alignment. We conclude that our method's false-positive rate is robust with respect to mis-alignment of neutrally-evolving regions of the genome.

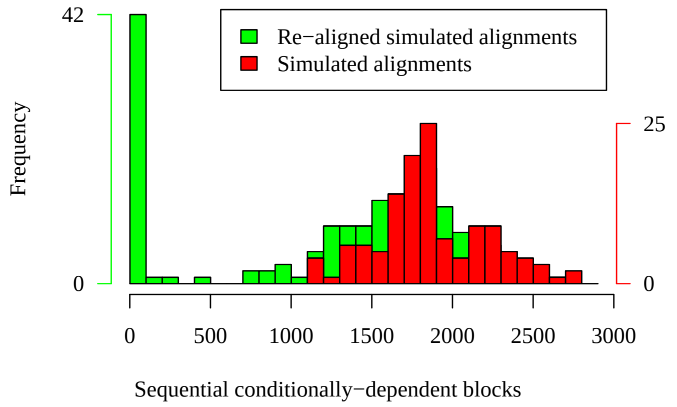

Figure 2. Distribution of the number of sequential conditionally-dependent blocks, indicating the number of overlapping or nested indel events, in the simulated data before and after re-alignment with `PECAN`. If the indel structure of the true (original) alignment was perfectly preserved, the two distributions would be identical.

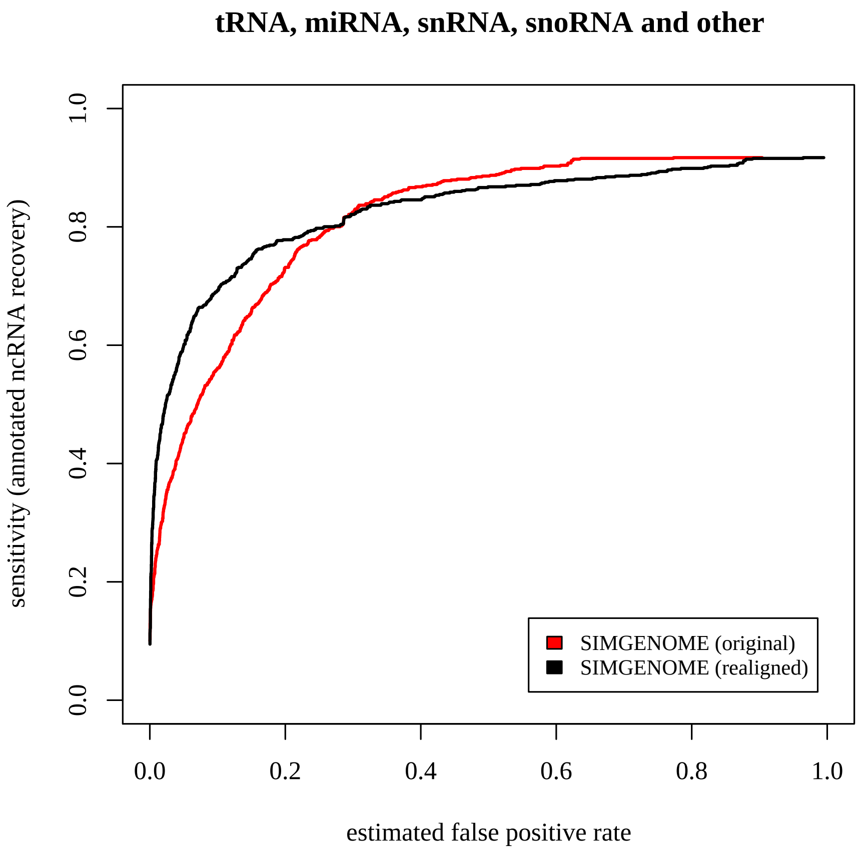

Figure 3. ROC curves for the ClosingBp grammar on simulated data (Indel2Lex3) before and after re-alignment with `PECAN`. Alignment error introduced by `PECAN` had surprisingly little effect on our estimated false-positive estimates, except near the high-specificity discovery threshold which we chose, where the estimated false-positive rate was up to 3-fold higher.

References

1. Wang A, Ruzzo W, Tompa M (2007) How accurately is ncRNA aligned within whole-genome multiple alignments? BMC Bioinformatics 8: 417.

2. Bray N, Pachter L (2004) MAVID: Constrained ancestral alignment of multiple sequences. Genome Research 14: 693-699.

3. Paten B, Beal K, Birney E (2008) Pecan: Large-scale consistency alignment. Accepted.

4. Holmes I, Durbin R (1998) Dynamic programming alignment accuracy. Journal of Computational Biology 5: 493-504.

5. Do CB, Mahabhashyam MSP, Brudno M, Batzoglou S (2005) ProbCons: Probabilistic consistency-based multiple sequence alignment. Genome Res 15: 330–340.

6. Schwartz AS, Pachter L (2007) Multiple alignment by sequence annealing. Bioinformatics 23: e24-9.

7. Alexandersson M, Cawley S, Pachter L (2003) SLAM: cross-species gene finding and alignment with a generalized pair hidden Markov model. Genome Research 13: 496-502.

8. Dewey CN (2007) Aligning multiple whole genomes with mercator and MAVID. Methods Mol Biol 395: 221-236.

9. Kim J, Sinha S (2007) Indelign: a probabilistic framework for annotation of insertions and deletions in a multiple alignment. Bioinformatics 23: 289–297.

10. Bradley RK, Holmes I (2007) Transducers: an emerging probabilistic framework for modeling indels on trees. Bioinformatics 23: 3258–3262.
